# Bladder capacity in an elimination communication infant: a case report

**DOI:** 10.1186/s13256-023-04267-4

**Published:** 2023-12-14

**Authors:** Roni Kraut

**Affiliations:** Department of Family Medicine, University of Alberta, 6-10 University Terrace, Edmonton, AB T6G 2T4 Canada

**Keywords:** First morning voided volume, Bladder capacity, Urinary bladder, Elimination communication, Assisted infant toilet training

## Abstract

**Background:**

Bladder capacity is essential in assessing children with voiding dysfunction, yet it is currently unclear how best to determine a benchmark bladder capacity; various formulas have been proposed.

**Case presentation:**

This report details the unique case of an elimination communication Caucasian infant (< 2 years old) who achieved nighttime and daytime dryness by 6 months of age. His first morning voids were measured from 8 to 20 months of age and compared with three formulas: (1) the Koff formula, a widely used formula based on fill volumes in anesthetized infants; (2) the Kaefer formula, a formula based on fill volume in nonanesthetized infants; and (3) the Holmdahl formula, a formula based on frequency–volume charts in normal infants.

**Conclusion:**

This infant’s first morning void was found to be most consistent with Kaefer's formula. Further research is required to determine if formulas based on fill volume in nonanesthetized infants provide the more accurate benchmark bladder capacity in infants.

## Background

An accurate estimate of bladder capacity, that is, the maximal fill volume of the bladder, is important diagnostically for children with voiding dysfunction. Bladder capacity is often determined indirectly through noninvasive frequency–volume charts (FVC), with the maximal voided volume (MVV) taken to approximate bladder capacity. It can also be determined directly through invasive procedures (for example, radionuclide cystography) or indirectly by first morning void (FMV), which is noninvasive and has been found to approximate the MVV > 70% of the time [1].

Bladder capacity formulas have been developed to provide bladder capacity benchmarks in the pediatric population, and there is currently equipoise on which formulas provide the best point of reference. The most widely used formula is a linear formula by Koff, based on cystometry findings of 35 anesthetized children undergoing surgery unrelated to the bladder [2]. Many other linear formulas have been proposed and often provide different benchmarks depending on whether they are based on a maximal fill volume (determined by a procedure) or based on FVC (including or excluding the FMV).

This case report is of a Caucasian infant (< 2 years old) who achieved nighttime and daytime dryness by 6 months of age with elimination communication, which is a toileting approach little known in the West that that involves caregivers being attentive and responding to infant cues. This presented a select opportunity to assess bladder capacity with the FMV and to compare bladder capacity with commonly used bladder capacity formulas. We selected three formulas: [2] Koff 1983, based on cystometry in anesthetized infants; [3] Kaefer 1997, based on radioactive cystography in nonanesthetized children; and [4] Holmdahl 1996, based on FMV in infants.

## Case presentation

This Caucasian male was born in 2017, full-term and healthy. He was toileted using elimination communication. He was given the opportunity to void many times during the day based on his signs and timing, and he rarely wore a diaper.

During his first month, he achieved daytime dryness around 50% of the time and was nearly 100% continent for his bowel movements. By the end of the first month, he slept in 3-hour increments and was dry throughout. By 4 months, he achieved daytime dryness around 60% of the time and slept up to 6 hours a night remaining dry. By 6 months, he was dry nearly 75% of the time during the day and 100% dry at night (sleeping on average 7 hours at a time). At 10 months, he started daycare, and he rarely had any accidents in any of the three daycares he attended. By 15 months, he had on average 10 hours of uninterrupted sleep a night, which is consistent with pediatric recommendations [5].

His FMV was measured starting at 8 months, as he provided a unique opportunity to assess bladder capacity (it is seldom possible to use FMV to approximate bladder capacity in infants, as nighttime dryness is on average achieved between 3 and 4 years old [6]). His FMV was measured 57 times from 8 to 20 months with a standard plastic measuring cup (markings every 50 ml) and estimated to the nearest 25 ml. His urine was poured from the potty to the plastic measuring cup with minimal spillage; in the uncommon event of substantial spillage, the sample was discarded. The volume of his FMV increased from a maximum of 75 ml at 8 months to 150 ml at 20 months. See Table 1 for these measurements along with maximum bladder capacity calculated with the Koff, Kaefer, and Holmdahl formulas. His maximum bladder capacity most closely approximates Kaefer’s formula. There were also 12 measurements taken of his void after naps (1–3 hours in duration) between 8 and 15 months, and the volume was always 50 ml.

**Table 1:** Bladder capacity and comparison with formula estimates

Age (months)	Data points	Weight (lbs)	Height (inches)	Median hours dry before voiding (IQR)	Median volume (IQR) (ml)	Max volume (ml)	Koff 1983^2^ (ml)	Kaefer 1997^3^ (ml)	Holmdahl 1996^4^ (ml)
8	9	20.6	27	7.0 (6.5–8.0)	75 (50–100)	100	80	100	58
9	5	21.4	28	7.0 (7.0–7.0)	100 (80–100)	100	83	105	61
10	2	–	–	9.0 (9.0–9.0)	75 (75–75)	70	85	110	63
11	6	21.8	28	7.0 (7.0–7.0)	85 (55–100)	100	88	115	66
12	4	23.2	29	8.5 (7.88–9.38)	87.5 (75–113)	150	90	120	68
13	5	–	–	7.0 (7.0–9.0)	50 (50–100)	100	93	125	71
14	3	23.7	30	8.5 (8.0–8.75)	50 (38–100)	150	95	130	73
15	6	–	–	10.3 (9.63–10.5)	62.5 (50–75)	100	98	135	76
16	7	23.1	31	10.0 (9.75–10.5)	150 (150–150)	175	100	140	78
17	3	–	–	11.0 (10.5–11.0)	150 (125–150)	150	103	145	81
18	–	–	–	–	–	–	105	150	83
19	6	24.1	33	9.5 (8.38–9.5)	125 (106–144)	150	108	155	86
20	1	–	–	10.0 (NA)	150 (NA)	150	110	160	88

## Discussion and conclusion

To our knowledge, this is the first study using FMVs of a child under 2 years old [1]. The study found that, similar to FMV in children greater than 3 years old, FMV in children under 2 years old may also provide a reasonable approximation of bladder capacity. It further found the formula by Koefer was most consistent with the bladder capacity of this infant.

Quantifying bladder capacity was initiated by Barbara Starfield in 1967 with a prospective cohort study in which she had both enuretic and nonenuretic children drink an oral water load (30 ml/kg of body weight) and estimated their functional bladder capacity based on the volume of the larger of their first two voided specimens (the specimen needed to be at least half of the oral water load) [7]. Since then, numerous linear formulas have been proposed to provide a benchmark for functional bladder capacity. The first formulas were based on how much fluid the bladder can hold in children undergoing invasive procedures (for example, radionucleotide cystography), while the more recent formulas are based on the noninvasive FVC; most of these formulas include the child’s age as the dependent variable [8].

The infant in this case report had an FMV that best agreed with the Kaefer formula (based on radioactive cystography in nonanesthetized children) and was higher than the benchmark bladder capacity based on the Koff and Holmdahl formulas. The Koff formula was developed based on 35 anesthetized children; the bladder of anesthetized infants may not provide normal bladder capacity. This is supported by a study by Rittig *et al.* on 148 children, which found that when FMV was included, the bladder capacity exceeded the estimate by Koss by 50–100 ml [9]. Further, the Holmdahl formula is based on FVC of infants not toilet trained, and even with including post-void residuals, it may not reflect actual bladder capacity. Cho *et al.* noted that for children with nighttime enuresis, FVC should be multiplied by a factor of 1.25 to accurately predict bladder capacity [1].

It was possible to use FMV to approximate bladder capacity in this case due to toileting with elimination communication. Elimination communication has been used over the world for hundreds of years, and it continues to be used in developing countries. It is different than the mainstream child-oriented approach that recommends waiting until a child is developmentally ready. Instead, it involves being attentive to their cues for eliminating, which is similar to being attentive to an infant’s cues for being tired and hungry. It allowed this infant to be “toilet trained” well before the expected time and appears to have a myriad of other benefits, including reduced diaper dermatitis, reduced colic [10], improved gait [11], and lower environmental burden [12].

There appears to be only one other study of bladder capacity of elimination communication infants; it found that daytime bladder capacity was close to the capacity predicted by Hjalma’s formula (estimated bladder capacity similar to the Holmdahl formula). However, bladder capacity was assessed during a 4-hour in-hospital observation period during the day, so it was unlikely to have included FMV [13].

This case report suggests that for infants, the formulas based on radionuclide cystography in nonanesthetized infants may provide the best approximated bladder capacity. It would be advantageous to have further studies on the bladder capacity of elimination communication infants to see if the results from this case report are reproducible and generalizable across different ethnicities.

## Data Availability

The dataset analyzed during the current study are available from the corresponding author on reasonable request.

## Abbreviations

FVC: Frequency–volume charts
MVV: Maximal voided volume
FMV: First morning void
